# Blood Groups and Breast Cancer

**DOI:** 10.12669/pjms.346.16824

**Published:** 2018

**Authors:** Anusha Sultan Meo

Worldwide, every year, October is celebrated to be the month for breast cancer awareness, where different societies and organizations aim to divert attention to this deadly disease. In the past, breast cancer whose existence was ignored, became the leading cause of several neglected deaths of females. Today, breast cancer stands as the most prevalent and deadly malignancy in the world and the second leading cause of cancer deaths in women^1^ affecting approximately 2.1 million females each year. Just in 2018, breast cancer took the lives of 627,000 women, accounting for 15% of all cancer deaths among females^2^.

The incidence of breast cancer has been allied with numerous factors including ageing, obesity, delayed childbearing, menopause, genetics, environment and diet^2^. However, new research and modern literature shows an association of breast cancer with blood groups. ABO and Rhesus blood groups are the major human blood group systems. Blood group system is associated with various diseases including gastric, duodenal ulcer, Hepatitis-B^3^, vascular diseases, abdominal aortic aneurism and type 2 diabetes mellitus^4^.

Statistics now reveal that Blood group “A” has the highest prevalence of breast cancer (45.8%) and blood group “AB” has a low (6.2%) incidence of breast cancer. Blood group “A+ve” has a high risk while blood type “AB-ve” has a low peril of breast cancer. Although the largest percentage of the global population has the blood group O+, the largest occurrence of breast cancer is of individuals with the blood group A+^5^.

To combat this increasing breast cancer incidence, screening programs in women are highly essential. Physicians must monitor females especially with blood group “A+ve” as these females are particularly prone to breast cancer. Moreover, other preventive measures include a healthy diet intake such as the usage of fruits and vegetables, regular exercise routine, maintaining a normal body weight, avoiding smoking, alcohol, food additives and oral contraceptive pills. All these physiological measures if exercised, will be a vital step in helping us win the war against breast cancer.

## References

[1] Anderson BO (2014). Breast cancer:thinking globally. Science.

[2] WHO Breast cancer.

[3] Liu J, Zhang S, Liu M, Wang Q, Shen H, Zhang Y (2018). Distribution of ABO/Rh blood groups and their association with hepatitis B virus infection in 3.8 million Chinese adults:A population-based cross-sectional study. J Viral Hepat.

[4] Meo SA, Rouq FA, Suraya F, Zaidi SZ (2016). Association of ABO and Rh blood groups with type 2 diabetes mellitus. Eur Rev Med Pharmacol Sci.

[5] Meo SA, Suraya F, Jamil B (2017). Association of ABO and Rh blood groups with breast cancer. Saudi J Biol Sci.

